# The Influence of User Characteristics and a Periodic Email Prompt on Exposure to an Internet-Delivered Computer-Tailored Lifestyle Program

**DOI:** 10.2196/jmir.1939

**Published:** 2012-03-01

**Authors:** Francine Schneider, Liesbeth van Osch, Daniela N Schulz, Stef PJ Kremers, Hein de Vries

**Affiliations:** ^1^CAPHRIDepartment of Health PromotionMaastricht UniversityMaastrichtNetherlands; ^2^NUTRIMDepartment of Health PromotionMaastricht UniversityMaastrichtNetherlands

**Keywords:** Internet interventions, computer tailoring, lifestyle, behavior change, program use, user characteristics, diffusion, proactive strategy, prompts

## Abstract

**Background:**

The Internet is a promising medium in the field of health promotion for offering tailored and targeted lifestyle interventions applying computer-tailored (CT) techniques to the general public. Actual exposure to CT interventions is not living up to its high expectations, as only a (limited) proportion of the target group is actually using these programs.

**Objective:**

To investigate exposure to an Internet-delivered, CT lifestyle intervention, targeting physical activity, fruit and vegetable intake, smoking behavior, and alcohol intake, we focused on three processes: first use, prolonged use, and sustained use. The first objectives were to identify user characteristics that predict initiation of an online CT lifestyle program (first use) and completion of this program (prolonged use). Furthermore, we studied the effect of using a proactive strategy, consisting of periodic email prompts, on program revisits (sustained use).

**Methods:**

The research population for this study consisted of Dutch adults participating in the Adult Health Monitor, offered by the regional public health services. We used a randomized controlled trial design to assess predictors of first use, prolonged use, and sustained use. Demographics and behavioral characteristics, as well as the strategy used for revisiting, were included as predictors in the model.

**Results:**

A total of 9169 participants indicated their interest in the new program and 5168 actually logged in to the program. Participants significantly more likely to initiate one of the CT modules were male, older, and employed, and had a lower income, higher body mass index, and relatively unhealthy lifestyle. Participants significantly more likely to complete one of the CT modules were older and had a higher income and a relatively healthier lifestyle. Finally, using a proactive strategy influenced sustained use, with people from the prompting condition being more likely to revisit the program (odds ratio 28.92, 95% confidence interval 10.65–78.52; *P* < .001).

**Conclusions:**

Older, male, and employed participants, and those with a lower income, higher body mass index, and a relatively unhealthy lifestyle were more likely to initiate a CT module. Module completers predominantly had a higher income and age. The current program therefore succeeded in reaching those people who benefit most from online lifestyle interventions. However, these people tended to disengage from the program. This underlines the importance of additional research into program adjustments and strategies that can be used to stimulate prolonged program use. Furthermore, sending periodic email prompts significantly increased revisits to the program. Though promising, this effect was modest and needs to be further examined, in order to maximize the potential of periodic email prompting.

**Trial Registration:**

Nederlands Trial Register (NTR: 1786) and Medical Ethics Committee of Maastricht University and the University Hospital Maastricht (NL2723506809/MEC0903016); http://www.trialregister.nl/trialreg/admin/rctview.asp?TC=1786 (Archived by WebCite at http://www.webcitation.org/65hBXA6V7)

## Introduction

With a substantial number of people accessing the Internet in search of health-related information [1,2], the Internet has developed into a popular medium for offering a broad range of specific health information, such as information on health and illness, details on treatment-related issues, and information on health promotion practices, such as programs that offer information and advice on lifestyle behaviors [3]. Since Internet penetration rates are still expanding, with almost two billion people having access to the Internet [4], the number of health-related searches is also expected to increase. The Internet is therefore considered to be a promising medium in the field of health promotion for offering tailored and targeted promotion programs to the general public [4-7]. As online health-promoting applications provide many opportunities for interactivity, they are particularly suited for implementing interventions that offer immediate feedback and advice to users. In particular, lifestyle interventions applying computer-tailored (CT) techniques [5,6,8], addressing health behaviors such as physical activity [9,10], fruit and vegetable intake [11,12], smoking cessation [13-16], and alcohol consumption [17,18] have been shown to have positive effects on health behavior.

Despite these promising prospects, actual exposure to CT interventions is not living up to the high expectations [19-23], as only a (limited) proportion of the target group actually uses these programs [21]. Earlier studies defined exposure as pertaining to three different aspects: accessing the intervention (first use), engaging in the intervention content for a substantial period of time (prolonged use), and revisiting the intervention (sustained use) [19]. The level of first-time use of online interventions is generally low, with only small proportions of the potential target population actually accessing the intervention [19,24,25]. Levels of actual engagement in the intervention and of revisits to the intervention are even lower [26]. Since health behavior change is a complex process, actual change requires prolonged and sustained commitment to the program to enable optimal support during the change process. Intensive engagement in an intervention session allows processing of the intervention content and involvement in its effective components and is therefore essential [22,27,28]. Furthermore, a specific number of repeated visits to an intervention may be imperative, as, due to a high dose–response relation [29,30], sustained use of the program is essential to further maximize its effect on subsequent health behavior change [31,32].

To increase adoption rates of online interventions, it is imperative to obtain detailed profiles of those who successfully adopt an intervention [24,25]. By closely studying characteristics of these first-time users and mapping how they use and reuse the intervention, detailed knowledge can be acquired on intervention adopters. It is important to reach those people most in need of online lifestyle interventions—that is, the people who engage in risky behaviors such as smoking, excessive alcohol use, lack of physical activity, or unhealthy eating patterns. Even though unhealthy lifestyle behaviors are prevalent among the whole population [33,34], studies have shown that those who have a lower income and educational level (ie, a low socioeconomic status) are generally more inclined to have an unhealthy lifestyle [34,35]. Furthermore, even though Internet access rates are increasing among people with a lower socioeconomic status [35], their actual exposure to Internet interventions is lagging [36-39]. Besides socioeconomic status, other user characteristics are reported to have an influence on adoption of online interventions. Previous studies have pointed out that Internet interventions tend to reach women [40-43] and older people [44]. It is important to gain more insight into characteristics of people who are being reached by the program, but also of people who are left unexposed to the program. These insights can be used to acquire knowledge on the development of further steps to improve exposure to the program.

Besides focusing on first use of the program, it is also imperative to optimize prolonged use of the program and to study related determinants. Previous studies indicated that tailoring in itself is a strategy to prevent early disengagement from online behavior change programs [28,45,46]. Furthermore, specific user characteristics such as age, gender, and level of education are related to level of program engagement [28,30,40], with women, older people, and those who are more highly educated displaying higher levels of program engagement. Since engagement in the program is an important predictor of revisits to the program [46], it is important to map how the program is used and to study determinants of prolonged program use. This obtained knowledge can be used to make specific program adjustments, or to develop specific strategies that can be used to enhance prolonged use of the program.

Online interventions are often offered reactively to the public, implying that a passive approach is used, in which users themselves must act in order to repeatedly benefit from the intervention [47]. However, since attaining visitors’ loyalty to an intervention over an extended period of time is a very strenuous process [22,23,30], efforts should be put into ensuring sustained use by employing more proactive strategies [27]. The use of periodic email prompts has been proposed as an effective proactive strategy to boost revisits to interventions aimed at stimulating a healthy lifestyle [48]. However, most studies merely explored the effectiveness and efficacy of the whole intervention, instead of focusing on the added value of periodic prompting as a separate intervention component. As a consequence, there is too little evidence on the absolute effectiveness of sending periodic prompts.

 In the present study, we aimed at answering three questions. First, which user characteristics predict initiation of an online CT program (first use)? Second, which user characteristics predict completion of the online CT program (prolonged use)? Third, what is the effect of using a proactive strategy, consisting of periodic email prompts, on program revisits (sustained use)? We addressed these questions among participants in an online CT intervention aimed at multiple health behaviors: physical activity, fruit and vegetable intake, smoking cessation, and alcohol consumption.

## Methods

In this randomized controlled trial (RCT) we compared the effect on program revisit of a proactive technique applying a periodic email prompt versus the use of a reactive approach. Levels of use and reuse of the program within a 4-month period were studied and linked to specific user characteristics. The current RCT was part of a larger RCT testing the effect of the CT program compared with a control group [49]. Only people allocated to the study arm receiving computer tailoring of the larger RCT were included in the current RCT. This study was approved by the Medical Ethics Committee of Maastricht University and the University Hospital Maastricht (NL27235.068.09/MEC 09-3-016) and is described in more detail elsewhere [49,50].

 The research population for this study consisted of Dutch adults of the provinces of Zeeland and North-Brabant, which participated in the Adult Health Monitor 2009 [51]. This Monitor is used by all regional public health services and takes place every 4 years. It serves as a monitoring tool to assess the overall level of health in the Dutch population by approaching a representative sample of the population to fill out a questionnaire assessing different aspects of general health (eg, physical and mental health) and health-related topics (eg, social and physical environment). Participants had the opportunity to complete a written or an online version of this monitoring questionnaire. The CT program was embedded in the online version and was offered as an additional service to online respondents. Data for the present study were collected from November 2009 to August 2010.

### Procedure and Respondents

After completing the online version of the Adult Health Monitor, all participants were introduced to the program and were offered the opportunity to receive, free of charge, CT feedback about their current health behaviors, such as physical activity, fruit and vegetable intake, alcohol consumption, and smoking. This program consisted of several modules, one per behavior, which incorporated questionnaire items and provided feedback on several sociocognitive determinants of each health behavior. The content of the program modules was based on programs that have been proven to be effective in RCTs for increasing smoking cessation, promoting the intake of fruit and vegetables, increasing the level of physical activity, and reducing the consumption of alcohol [17,51-54].

Participants who were interested in the new program were asked to leave their email addresses. They received an email including an invitation to log in to the CT program with a personal log-in code and password, approximately 3 weeks after completion of the Monitor. By logging in to the program, participants received detailed information on the content and purpose of the study. Subsequently, data on demographics and the five health behaviors obtained through the Monitor were transported to the CT program, resulting in a personal overview of individuals’ current health behavior status. If respondents were not adhering to the Dutch public health guidelines set for these behaviors, a module generating CT health advice for changing behavior was available for each health behavior.

During a 4-month period after the baseline visit, we monitored use of the intervention. People in the prompting condition were prompted proactively via email 3 months after their baseline visit to revisit the CT program. Revisits to the program were stimulated to provide participants with the opportunity to monitor their own behavior. During a revisit participants could log in to the program and complete the health risk appraisal questionnaire. Based on their answers, a new personal health overview was composed entailing information on their current health behavior status, as well as on their status during all previous visits. Improvements, deteriorations, or stability of health behaviors were graphically presented. People in the no-prompting condition did not receive any additional prompts and were encouraged only at baseline to revisit the program. Reactions to this email prompt were monitored during a 1-month period.

### Randomization

All included participants were randomly allocated to a prompting condition (receiving additional email prompts) or a no-prompting condition (receiving no additional email prompts). We used a computer software randomization device to determine random allocations at the respondent level.

### Content

#### CT Program

The CT program used a dual approach to guide people toward behavior change. The first part consisted of a health risk appraisal and was aimed at increasing participants’ *awareness* of their health behavior status, by comparing their status to the Dutch public health guidelines set for these health behaviors (ie, being moderately physically active for 30 minutes at least 5 days a week, eating two pieces of fruit per day, eating 200 g of vegetables per day, not drinking more than one [women] or two [men] glasses of alcohol a day, and not smoking). In this health risk appraisal, feedback messages were used to inform people of their status for each health behavior and to provide them with additional information on the content of the separate guidelines. These feedback messages were complemented by using graphic representations of traffic lights [55], with a green light corresponding to adherence to the guidelines and a red light corresponding to nonadherence. An amber light was used for people who were close to adherence to these guidelines. In case of discrepancies between current behavior and the guidelines, people were alerted and directed to the CT modules.

 Second, assistance was provided in *changing* participants’ health *behavior* by offering five separate CT modules. The content of these modules was based on the Integrated Model for exploring motivational and behavioral change (I-Change Model) [56]. The modules used a fixed, gradual approach consisting of four steps, guiding people toward behavior change. The first step addressed the pros and cons of engaging in the desired behavior under consideration. The second step focused on the role of significant persons in the direct environment and strategies on how to deal with lack of support and bad role models. The final two steps used planning strategies and were aimed at helping people form preparatory plans to start changing their behavior (step 3) and coping plans to help them overcome difficult situations and prevent relapse (step 4) after changing their behavior. Within the modules, all health advice was adapted to individuals’ characteristics by considering demographic, behavioral, and cognitive characteristics [57-59]. Demographic and behavioral characteristics, such as participants’ gender and health behavior status, were directly obtained through the Adult Health Monitor. Cognitive variables, such as attitude, perceived social influence, self-efficacy, intention, and planning strategies (action plans and coping plans), were assessed by using an additional tailoring questionnaire.

The CT modules were embedded in a website (http://www.mijngezondgedrag.nl/) that was especially designed for the current project. This website contained general information considering a healthy lifestyle and the selected health behaviors. Furthermore, the website provided specific information regarding the project, a direct link to the CT program, and information on frequently asked questions. During the study, new information (eg, advice-supporting messages, recipes, and facts) was structurally added to the website.

#### Email Prompt

Participants in the prompting condition received an email 3 months after their baseline visit, prompting them to revisit the program. This email opened with a personalized greeting and reminded people about their first visit to the program. Subsequently, people were invited to revisit the program to obtain information on their health status and to monitor their progress. Participants were also informed of the opportunity to receive additional, iterative health advice on the health behavior(s) selected at baseline or on a new behavior. Finally, to facilitate logging in to the program, the email also contained details on personal log-in information (user name and password). The email concluded with greetings from the research team and contact information.

### Measures

We collected user characteristics to produce a detailed user profile including information on personal characteristics, health behavior status, and intention to change their health behaviors.

#### Personal Characteristics

Questions pertaining to personal characteristics included questions on age, gender, educational level, personal net monthly income, work situation, marital status, and native country (Table 1).

**Table 1:** Characteristics of visitors to an online computer-tailored lifestyle program (n = 3448).

Demographic characteristic			Data
**Age (years)**			
	Range		19–64
	Mean (SD)		43.61 (12.60)
**Gender, n (%)**			
	Male		1822 (52.8%)
	Female		1626 (47.2%)
**Education level, n (%)**			
	Low		744 (21.6%)
	Medium		1188 (34.45%)
	High		1452 (42.11%)
	Unknown		64 (2%)
**Personal net monthly income (€), n (%)**			
	<1000		223 (6.5%)
	1001–1350		227 (6.6%)
	1351–1750		367 (10.6%)
	1751–3050		1174 (34.05%)
	>3051		966 (28.0%)
	Unknown		491 (14.2%)
**Employment, n (%)**			
	Employed		2614 (75.8%)
	Unemployed		662 (19.2%)
	Unknown		172 (5.0%)
**Marital status, n (%)**			
	Married		2089 (60.6%)
	Living together		526 (15.3%)
	Unmarried		635 (18.4%)
	Divorced		170 (4.9%)
	Widowed		28 (0.8%)
**Native country, n (%)**			
	The Netherlands		3277 (95.0%)
	Other		171 (5.0%)
**Body mass index (kg/m2), n (%)**			
	<18.5		57 (1.7%)
	≥18.5 to <25		1795 (52.1%)
	≥25 to <30		1240 (36.0%)
	≥30		356 (10.2%)
**Behavioral characteristic, n (%)**			
	Physical activity		
		Compliant	2939 (85.2%)
		Noncompliant	509 (14.8%)
	Vegetable consumption		
		Compliant	1121 (32.5%)
		Noncompliant	2327 (67.5%)
	Fruit consumption		
		Compliant	1552 (45.0%)
		Noncompliant	1896 (55.0%)
	Smoking behavior		
		Compliant	2790 (80.9%)
		Noncompliant	658 (19.1%)
	Alcohol intake		
		Compliant	2467 (71.5%)
		Noncompliant	981 (28.5%)
**Number of guidelines complied with, n (%)**			
	0		25 (0%)
	1		203 (5.9%)
	2		677 (19.6%)
	3		1235 (35.82%)
	4		936 (27.1%)
	5		372 (10.8%)

#### Health Behavior Status

Health behavior status consisted of information regarding the five key behaviors. Physical activity was measured by the Short Questionnaire to Assess Health-Enhancing Physical Activity [60], and guideline adherence was calculated following procedures used by Ainsworth et al [61]. Fruit consumption was measured by using a 4-item food frequency questionnaire assessing weekly fruit and fruit juice intake [62], and vegetable consumption was measured using a 4-item food frequency questionnaire assessing the weekly amount of consumed boiled or baked vegetables as well as salad or raw vegetables [62]. The consumption of alcohol was measured by the Dutch Quantity-Frequency-Variability Questionnaire [63]. And finally, smoking status was assessed by asking participants whether they smoked, what they smoked (cigarettes, cigars, packets of pipe tobacco) and how much they smoked per day (cigarettes) and per week (cigars or packets pipe tobacco).

#### Body Mass Index

Data on participants’ height (in centimeters) and weight (in kilograms) were used to calculate their body mass index (BMI). BMI is a heuristic used to estimate the level of body fat, and it is defined by dividing a participant’s body weight by the square of the participant’s height.

#### Intention to Change

Intention to change a health behavior was measured by means of 1 item using an algorithm consisting of 10 stages varying from unawareness to action. This variable was recoded in accordance with an adjusted version of the stages of change concept: immotivation (1), precontemplation (2), contemplation (3), preparation (4), and action (5) [64].

#### Program Evaluation

After completion of each single module, visitors were asked to evaluate the module by providing an overall grade (1 to 10).

### Outcomes

To measure first use of the program, we created a dichotomous variable based on program monitoring data, indicating whether participants initiated a module. Initiating a module was labeled as yes when people filled out the first question of this module. To measure prolonged use, we created a new dichotomous variable to indicate whether participants finished a module. Completion of a module was labeled as yes when people also filled out the final question of the module.

 To establish sustained use of the program, we created another dichotomous variable, indicating whether participants logged in to the program after baseline. Revisiting of the program was measured by comparing the dates of log-in with the baseline date.

### Statistical Analysis

First, we used general descriptive statistics to describe personal characteristics of the participants, as well as the main findings concerning current health behavior adherence to the public health guidelines. Second, logistic regression analyses were conducted. Initiation of a module and completion of a module (0 = no/1 = yes) were the dependent variables. Demographics (age, gender, marital status, native country, educational level, work status, and income), BMI, and health behavior status were included in the model as predictors of initiation. We included the same variables, with addition of intention (measured at the beginning of each CT module), in the model as predictors of completion. Logistic regression analysis was conducted with revisiting of the program (0 = no/1 = yes) as the dependent variable. The same demographics, as well as study condition, program evaluation, and initiation and completion of a module, were included in the model as predictors of revisiting of the program. All statistical analyses were done in SPSS version 7.0 (IBM Corporation, Somers, NY, USA).

## Results

### Site Visitors’ Baseline Characteristics

In total, 3448 people were allocated to the study arm receiving computer tailoring of the larger RCT and were included in the current RCT. Of all participants, with a mean age of 44 (SD 12.60) years, a little more than half were men, and most had a medium to high education level and an average to high monthly income (Table 1). Three-quarters of all participants were employed and more than half were married. Approximately 2% (57/3448) of all visitors were underweight, whereas more than half had a normal weight. One-third of visitors were slightly overweight and 10.3% (356/3448) were obese. A randomization check revealed no significant differences between respondents in the prompting and no-prompting condition.

Regarding the five health behaviors included in the program, 14.8% (509/3448) did not comply with the Dutch guidelines of at least 30 minutes of moderately intensive physical activity at least 5 days a week. With regard to fruit and vegetable intake, respectively 54.99% (1896/3448) and 67.49% (2327/3448) were not adhering to the Dutch guidelines of at least two pieces of fruit and at least 200 g of vegetables each day. About one in five visitors indicated to smoke 19.1% (658/3448), and a little more than a quarter (981/3448, 28.5%) did not comply with the Dutch guidelines for alcohol intake.

### First Use of the Program

#### CT Module Initiation

**Figure 1:**
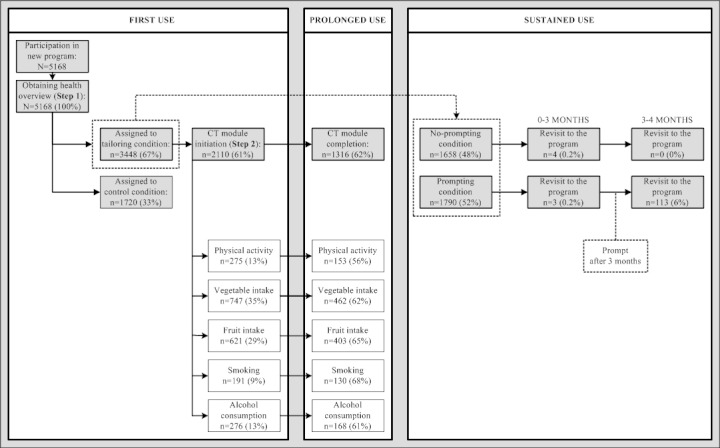
Flowchart exposure to CT program: first use, prolonged use and sustained use.

A total of 1338 participants (38.81%) did not participate in the second part of the program, leaving after receiving their personal health overview. The remaining 2110 visitors (61.19%) decided to obtain CT lifestyle advice by initiating one of the five behavioral modules. Of all the visitors who decided to obtain personalized health advice, 13.0% (n = 275) initiated the physical activity module, 35.4% (n = 747) and 29.4% (n = 621) initiated the vegetable and fruit modules, respectively, 9.1% (n = 191) initiated the smoking module, and 13.1% (n = 276) initiated the alcohol module (Figure 1).

#### Predictors of CT Module Initiation

Results of multiple logistic regression analyses indicated that male, older, and employed participants, and those with a lower income and a higher BMI were more likely to initiate one of the CT modules (Table 2). Initiation of a CT module was related to the total number of guidelines people adhered to: those with a relatively unhealthy lifestyle (fewer guidelines complied with) were more likely to initiate one of the CT modules (odds ratio [OR] 0.84, 95% confidence interval [CI] 0.77–0.90; *P* < .001).

**Table 2:** Results of multivariate logistic regression analyses of demographic characteristics, behavioral characteristic, and intention, with initiating a behavioral computer-tailored module as dependent variable (n = 3448).

Characteristic			Initiation of a behavior change module		
			OR^a^	*P* value	95% CI^b^
**Demographic characteristics**					
	Age		1.35*	0.00	1.22–1.49
	Gender				
		Female (reference)	1.00		
		Male	1.38*	0.00	1.16–1.64
	Education level				
		High (reference)	1.00		
		Low	1.07	0.59	0.83–1.38
		Medium	1.11	0.31	0.91–1.34
	Income		0.46*	0.00	0.42–0.52
	Employment				
		Unemployed (reference)	1.00		
		Employed	1.16*	0.03	1.01–1.33
	Marital status				
		Not in a relationship (reference)	1.00		
		In a relationship	1.00	1.00	0.79–1.28
	Native country				
		Other (reference)	1.00		
		The Netherlands	1.38	0.12	0.92–2.07
	Body mass index		1.03*	0.02	1.01–1.05
**Behavioral characteristic**					
	Number of guidelines complied with		0.84*	0.00	0.77–0.90

^a^ Odds ratio.

^b^ Confidence interval.

*Significant association (*P* < .05).

### Prolonged Use of the CT Program

#### CT Module Completion

Of all the visitors who initiated one of the CT modules to obtain personalized health advice (n = 2110), 62.37% completed a module (n = 1316). A total of 153 of 275 visitors completed the physical activity module (55.6%), 462/747 (61.9%) and 403/621 (64.9%) completed the vegetable and fruit modules, respectively, 130/191 (68.1%) completed the smoking module, and 168/276 (60.9%) completed the alcohol module (Figure 1). All modules were evaluated by visitors upon completion. The physical activity module was graded 7.09 (SD 1.41), vegetable consumption 7.45 (SD 1.02), fruit consumption 7.33 (SD 1.29), smoking 7.35 (SD 1.11), and alcohol consumption 7.39 (SD 1.11).

#### Predictors of CT Module Completion

Results of multiple logistic regression analyses indicated that older participants and participants with a higher income were more likely to complete one of the CT modules (Table 3). Participants with a relatively healthy lifestyle (complying with more guidelines) were also more likely to complete one of the CT modules.

**Table 3:** Results of multivariate logistic regression analyses of demographic characteristics, behavioral characteristic, and intention, with completion of a behavioral computer-tailored module as dependent variable (n = 2110).

Characteristic			Completion of a behavior change module		
			OR^a^	*P* value	95% CI^b^
**Demographic characteristics**					
	Age		1.49*	0.00	1.33–1.67
	Gender				
		Female (reference)	1.00		
		Male	1.05	0.63	0.86–1.29
	Education level				
		High (reference)	1.00		
		Low	1.06	0.67	0.80–1.41
		Medium	1.02	0.89	0.81–1.28
	Income		1.30*	0.00	1.17–1.45
	Employment				
		Unemployed (reference)	1.00		
		Employed	0.90	0.12	0.78–1.03
	Marital status				
		Not in a relationship (reference)	1.00		
		In a relationship	0.89	0.38	0.68–1.16
	Native country				
		Other (reference)	1.00		
		The Netherlands	0.87	0.54	0.57–1.34
	Body mass index		1.00	0.94	0.97–1.03
**Sociocognitive characteristic**					
	Intention				
		Action (reference)	1.00		
		Immotivation	1.03	0.85	0.77–1.37
		Precontemplation	1.34	0.41	0.67–2.72
		Contemplation	0.94	0.77	0.62–1.42
		Preparation	1.03	0.83	0.78–1.36
**Behavioral characteristic**					
		Number of guideline complied with	1.28*	0.02	1.02–1.24

^a^ Odds ratio.

^b^ Confidence interval.

^c^ Coded as 0 (no compliance) or 1 (compliance).

*Significant association (*P* < .05).

### Sustained Use of the CT Program

Additional emails prompting a revisit to the program were sent to 51.91% of all participants (n = 1790), while 48.09% (n = 1658) did not receive an additional reminder. In total, 3.5% (n  =  120) of all visitors decided to revisit the program within a 4-month period after baseline. Among those in the prompting condition, 0.2% (n = 3) revisited the program within the first 3 months (*before* receiving an additional reminder). A total of 113 participants (6.3%) reacted to the email prompt after 3 months and revisited the program. This number was significantly higher than in the no-prompting condition that received no additional reminder (n = 0, 0.0%; OR 28.92, CI 10.65–78.52; *P* < .001).

 Since the number of people from the control condition who revisited the program was extremely low (n = 4), we conducted additional analyses on predictors of revisiting solely within the experimental condition. Univariate logistic regression analyses revealed that completion of a behavior change module significantly predicted revisiting the program (OR 2.58, CI 1.57–4.26; *P* < .001). However, after multivariate analyses, including all predictors, this effect became nonsignificant.

## Discussion

The current study aimed at investigating the level of exposure to a CT online lifestyle program integrated into the Dutch Adult Health Monitor. We addressed user characteristics that predicted initiation (first use) and completion (prolonged use) of the CT program and tested a proactive strategy using periodic email prompts to enhance revisits to the program (sustained use).

### First Use

Since people with a lower income and educational level are generally more inclined to have an unhealthy lifestyle [36,65], the current program aimed at reaching especially those people in need of lifestyle improvement. However, in line with previous studies, the majority of participants in our sample had a medium to high educational level, an average income, and a relatively healthy lifestyle [36-39]. Even though integrating the CT program into the Monitor environment provided an access point for reaching a substantial proportion of the research population through tailored lifestyle advice, it did not succeed in predominantly reaching the people who benefit most from CT interventions.

 However, taking a closer look at CT module initiation reveals that initiators were significantly older, and likelier to be male, employed, and to have a lower income. Furthermore, participants with a higher BMI and an unhealthier lifestyle were more likely to start with one of the CT modules. Whereas the findings concerning age and gender are largely comparable with those of earlier studies [28,30,38,40], the present results are in contrast to previous findings that online health promotion programs tend to be used predominantly by people with a high socioeconomic status and healthy lifestyle [28,30,38,40].

The results imply that the current program succeeded in stimulating those people who are expected to benefit most from lifestyle interventions (ie, the people who engage in risky behaviors, but also people from specific risk groups such as older people and obese people) to initiate one of the CT modules. Embedding the CT program in an existing context, the Adult Health Monitor, may account for these findings. The main objective of the Monitor is to assess different aspects of general health and health-related topics, such as curative care, environment and everyday surroundings, social environment, social safety and violence and nursing and care. Due to this simple focus on assessment and not on health behavior change, participation may have been appealing to participants who are not primarily interested in behavior change. However, direct transportation of data from the Monitor to the CT program database allowed for the immediate and effortless composition of a personal health overview. Subsequently, this overview may have increased awareness of people’s lifestyle status and might have served as a cue to action to change their lifestyle, explaining the increased interest in the CT modules, among the unhealthy and low-socioeconomic status participants.

### Prolonged Use

Even though the CT program succeeded in attracting people with a relatively unhealthy lifestyle and low-socioeconomic status people to initiate one of the CT modules, it fell short in actually engaging these visitors, since module completers predominantly had a higher income, which is in line with previous studies [28,30,38,40]. The suboptimal level of interactivity within the CT program might account for these findings. Interactivity has been defined as the degree of 2-way interaction that is provided in the program [66,67], providing options for either immediate feedback or reciprocal interaction. Even though the program provided immediate feedback consisting of personalized health information, it did not provide communication features such as email contact and a discussion board, or features such as videos and games [68].

 Effort should therefore be put into adjusting program components and content to the needs and wishes of this target group and into developing strategies to keep low-socioeconomic status participants engaged in the program.

### Sustained Use

Our results from the current study indicate that prompting visitors to revisit the CT program is an effective strategy to enhance sustained use. However, the effect was limited, with only small percentages of people actually responding to the prompt. Replicating this study is therefore desirable. A possible explanation for these results might be the level of tailoring in the prompt. Although the content of the prompt was tailored to user characteristics, the level of personalization may have been too low, making the prompt less appealing and relevant. Furthermore, since the first prompt was sent after 3 months, people may have forgotten about the program and their participation in it.

The added value of the email prompts used in the present study could be further augmented. Several studies have suggested that the effect of email prompts increases if they alert people to new content that is provided on the program website [69] and if they provide information that is perceived as personally relevant [48,70]. Furthermore, the time of week when prompts are sent influences their effect, with prompts being sent at the beginning of the week being more effective [70].

Since email is a very popular online function used by substantial number of Internet users [71], it is a promising tool to attract people to health-promoting interventions. However, a potential downside of its popularity is the frequent use of email for marketing purposes. Due to the increase in email advertisements, or spam, dissatisfaction among email users is increasing [71] and emails are often neglected or deleted before being read. Future studies should therefore aim at developing prompt content that attracts receivers’ attention amidst a plethora of emails sent for marketing purposes. Furthermore, the optimal frequency at which prompts are delivered should be studied to yield further knowledge on future refinements and to maximize their potential.

### Strengths and Limitations

This study focused on exposure to an online CT lifestyle program by mapping characteristics of first-time users, examining predictors of first, prolonged and sustained use and by testing the efficacy of a proactive strategy to increase sustained use of the program. Even though several studies have highlighted the added value of periodic email prompts [48], this study is one of the few to focus on the added value of periodic prompting by testing its effect in a randomized control trial.

However, this study is liable to several limitations that need to be accounted for when interpreting the results. First, we used one generic email to prompt revisits to the CT program. Future research should thoroughly study the optimal content of prompts, in order to make them stand out amidst advertisement emails and spam, and to make them more personally relevant and persuasive. Principles used in the field of e-marketing and e-commerce might provide essential information on specific strategies to increase the attractiveness of email prompts.

Finally, even though this study provides evidence for the effectiveness of prompts in enhancing program revisits, additional research is needed to study their effect on subsequent behavior change because, due to a high dose–response relation, multiple visits to the program are necessary to maximize its effect on health behavior.

### Conclusion

We found that the CT modules were significantly more often initiated by older, male, employed, low-income participants with a relatively unhealthy lifestyle, which implies that the program succeeded in reaching and engaging those people who benefit most from online lifestyle interventions. However, people with a lower income tended to disengage from the CT program before finishing the modules. This underlines the importance of additional research into specific program adjustment and strategies that can be used to stimulate prolonged program use by low-socioeconomic status visitors. Furthermore, sending periodic email prompts significantly increased revisits to the program. Though promising, this effect was modest and needs to be further examined, in order to maximize the potential of periodic email prompting.

## Abbreviations

BMI: body mass index
CT: computer tailored
